# mRNA/microRNA Profile at the Metamorphic Stage of Olive Flounder (*Paralichthys olivaceus*)

**DOI:** 10.1155/2011/256038

**Published:** 2011-04-19

**Authors:** Caixia Xie, Shanliang Xu, Linlin Yang, Zhonghe Ke, Jubin Xing, Junwei Gai, Xiaoling Gong, Liuxiong Xu, Baolong Bao

**Affiliations:** ^1^The Key Laboratory of Exploration and Utilization of Aquatic Genetic Resources, Shanghai Ocean University, Ministry of Education, Shanghai 201306, China; ^2^Key Laboratory of Marine Biotechnology, Ningbo University, Ningbo 315211, China; ^3^The Key Laboratory of Sustainable Exploitation of Oceanic Fisheries Resources, Shanghai Ocean University, Ministry of Education, Shanghai 201306, China

## Abstract

Flatfish is famous for the asymmetric transformation during metamorphosis. The molecular mechanism behind the asymmetric development has been speculated over a century and is still not well understood. To date, none of the metamorphosis-related genes has been identified in flatfish. As the first step to screen metamorphosis-related gene, we constructed a whole-body cDNA library and a whole-body miRNA library in this study and identified 1051 unique ESTs, 23 unique miRNAs, and 4 snoRNAs in premetamorphosing and prometamorphosing *Paralichthys olivaceus*. 1005 of the ESTs were novel, suggesting that there was a special gene expression profile at metamorphic stage. Four miRNAs (*pol-miR-20c*, *pol-miR-23c*, *pol-miR-130d*, and *pol-miR-181e*) were novel to *P. olivaceus*; they were characterized as highly preserved homologies of published miRNAs but with at least one nucleotide differed. Representative 24 mRNAs and 23 miRNAs were quantified during metamorphosis of *P. olivaceus* by using quantitative RT PCR or stem-loop qRT PCR. Our results showed that 20 of mRNAs might be associated with early metamorphic events, 10 of mRNAs might be related with later metamorphic events, and 16 of miRNAs might be involved in the regulation of metamorphosis. The data provided in this study would be helpful for further identifying metamorphosis-related gene in *P. olivaceus*.

## 1. Introduction

Flatfish is famous for the asymmetric transformation during metamorphosis, especially one eye migrating to the other side. Other metamorphosis events include cranium deformation, asymmetric pigmentation, and 90-degree rotation in posture with a lifestyle transition from pelagic to benthic. The molecular mechanism of morphologic left/right asymmetry in Olive flounder, *Paralichthys olivaceus*, was thought to be different from that of interior organ asymmetry in vertebrate [1]. Thyroid hormone (TH) was proposed to regulate metamorphosis in flatfish [2–5]. As the nuclear receptor of TH, thyroid hormone receptor (TR) should be involved in the TH-inducing signal pathway. The spatial expression of the TR genes has been investigated in the metamorphosing Olive flounder [6]; however, it still cannot determine which metamorphosis events were regulated by TR in flatfish. In TH-TR signal pathway, the downstream genes will unavoidably be investigated in the future. To date, very few genes were investigated in metamorphosing flatfish [6–9]. Even though the cDNA libraries of various tissue types in *P. olivaceus* were constructed, especially immune-related tissues [10], the gene expression profile in metamorphosing *P. olivaceus* was still unavailable. 

Expressed sequence tags (ESTs) analysis is an efficient approach to characterize transcriptome. Large-scale EST sequencing project as a part of genome project has been conducted for several teleost species, such as salmonid and catfish [11–13]. Small-scale ESTs analysis has also been carried out for some aquaculture teleosts [10, 14–16]. In this study, we tried to enrich ESTs data and investigated the gene expression profile via cDNA library random sequencing in the premetamorphosing and prometamorphosing *P. olivaceus*. In addition, Johnston and Hobert reported that one microRNA termed lsy-6 controlled neuronal left/right asymmetric expression of chemosensory receptor in *Caenorhabditis elegans* [17]. Accordingly, the possible roles of microRNAs in regulating metamorphosis in flatfish should not be neglected. This is the reason that we constructed a microRNA library and analyzed its expression profile in the metamorphosing *P. olivaceus* in this research as well.

## 2. Material and Methods

### 2.1. Fish Maintenance and Sampling

Larvae were obtained from the Central Experiment Station of Chinese Academy of Fisheries Sciences (Beidaihe, Hebei, China) and then transported to the laboratory in Shanghai Ocean University, Shanghai, China. The larvae were reared in the laboratory according to the methods provided in [8]. Larvae were fed live brine shrimp (*Artemia*) nauplii until the end of metamorphosis. We use the following classifications for the metamorphic stages of *P*. *olivaceus* in this study [18, 19]: Premetamorphosis (17 DAH, days after hatching), the stage prior to the start of eye migration; Prometamorphosis (19 DAH), from the start of eye migration until the start of resorption of several elongated dorsal fin rays; Climax (23 DAH), from the start of resorption of the elongated dorsal fin rays until the completion of fin resorption and eye migration; Postclimax (27 DAH), after the completion of fin resorption and eye migration. All samples were frozen using liquid nitrogen and stored at −80°C until proceeding to total RNA isolation.

### 2.2. cDNA Library Construction and Sequencing

Total RNA was isolated from premetamorphosing or prometamorphosing larvae (17DAH and 19DAH) using TRIzol Reagent (Invitrogen, Carlsbad, Calif, USA) according to the manufacturer's instruction. Equal amounts of total RNA from premetamorphosing or prometamorphosing larvae were pooled. mRNA was purified from total RNA using Oligotex mRNA Kits (QIAGEN, Valencia, Calif, USA) according to the manufacturer's instruction. A directional cDNA library of the whole larvae was constructed using the pBlueScript II SK+ vector (Stratagene, La Jolla, Calif, USA). First strand cDNA was synthesized according to the protocol of superscript II RNase H-reverse transcriptase (Invitrogen). Oligo (dT)18 primer with *Xho* I digestion site was used for the synthesis of first cDNA strand. Second strand was synthesized using DNA polymerase I (Promega, Madison, Wis, USA). cDNAs 0.5–2 kb size of were inserted into pBluescript II SK+ vector and then were electroporated into competent cells. Over 5000 primary cDNA clones were obtained with an average insert size of >1 kb. Titer of the primary cDNA library was over 1 × 10^6^, and then it was amplified once before colonies were picked for sequencing (Biotecan, Shanghai, China). The vector sequence was trimmed from the EST sequences using Vector NTI suite 8.0 (Invitrogen). Trimmed sequences were further screened using the ContigExpress in Vector NTI suite 8.0. High-quality ESTs were then assembled into clusters of contiguous sequences (contigs). Vector NTI suite 8.0 was used for contig assembly using stringent parameters, that is, overlap length cutoff of 100 and overlap percent identity of 90. The consensus sequence of each contig and singletons comprising the unique sequences were sent to the National Center for Biotechnology Information (NCBI) by using online software Blast2go [20] to be compared against the nonredundant protein database using BLASTX. The E-value cutoff was *1e−5*. Novel ESTs were also identified by comparison with *P. olivaceus *EST sequences in dbEST at NCBI using BLASTN. All ESTs that were not identified as orthologs of known genes were designated as unknown EST clones.

Sequences with BLASTX hits were mapped and annotated according to gene ontology terms (GO) in AmiGO database (http://amigo.geneontology.org/cgi-bin/amigo/go. cgi). The distribution of genes in each of the main ontology categories was examined, and the percentages of unique sequences in each of the assigned GO terms were calculated. In each of the three main categories of GO, namely, biological process, molecular function, and cellular component [21], 100% was considered as the total number of unique sequences having an assigned GO term. Thus, in each main category, the percentages of 2nd level do not add up to 100% because some deduced proteins have more than one GO category assigned to them [22].

### 2.3. MicroRNA Library Construction and Sequencing

RNA with size less than 200 nt from premetamorphosing or metamorphosing larvae was isolated using mirVana miRNA Isolation Kit (Ambion, Austin, Tex, USA) following manufacturer's instructions with minor modifications. In brief, equal amounts of the above RNA from premetamorphosing or prometamorphosing larvae were pooled. 2 *μ*g of pooled RNA were polyadenylated at 3′ hydroxyl terminus by using poly(A) polymerase (New England BioLabs, Ipswich, Mass, USA) incubation for 15 min at 37°C. Then, the 5′ DNA/RNA linkers (5′-ACGGAAuuccucacuaaa-3′) were ligated to the 5′ end by T4 RNA ligase incubation for 1 h at 37°C. This mixture was then reverse transcribed by MMLV reverse transcriptase (Promega, Madison, Wis, USA) using primer complementary to the 3′ linker sequence (5′-CTAGCTTGGTGCCTGGAATTCGCGGTTTTTTTTTTTTTTTTTTTTTTTTTT) at 42°C for 1 h, and PCR was amplified using forward primer (5′-CCAACCGGCACCACGGAATTCCTCACTAAA) and reverse primer (5′-CTAGCTTGGTGCCTGGAATTCGCGGTTTTT) on both linkers. The reactions were completed with the following thermoprofiles: 95°C for 15 min for one cycle, then the samples were amplified for 35 cycles at 94°C for 1 min, 58°C for 30 s, and 72°C for 30 s. Upon the completion of PCR, the reaction was incubated at 72°C for additional 10 min. PCR products were analyzed by electrophoresis on a 12% nondenaturing polyacrylamide gel electrophoresis (PAGE). The bands from 235 bp to 245 bp were excised and purified. The purified PCR fragments were then ligated into pGEM-T Easy Vector (Promega) and transformed into the competent* DH5*α** cells. Transformed bacterial cells were plated and grown overnight. Then the colonies were picked and sequenced (Biotecan). Small RNA sequence data were analyzed by BLAST search against the miRBase database (http://www.mirbase.org/). MicroRNAs were identified and named based on sequence homology to published miRNAs according to the universal nomenclature [23].

### 2.4. Quantitative Real-Time Reverse Transcription Polymerase Chain Reaction

Total RNA from the whole larvae at different metamorphic stages was isolated using TRIzol reagent (Invitrogen) followed by DNase treatment. The abundance of mRNA or miRNA was quantified by qRT-PCR or stem-loop qRT-PCR [24], respectively. 2 *μ*g of DNase-treated RNA was converted to cDNA using MMLV reverse transcriptase (Promega). qRT-PCR primers for each mRNA are listed in Table 1 and for miRNA in Table 2. The relative expression of mRNA and miRNA was normalized using *β*-actin mRNA and U6 snRNA as control, respectively. For qRT-PCR or stem-loop qRT-PCR, the thermocycler was set at 95°C for 15 s and 55°C for 60 s per cycle for a total of 40 cycles, followed by 95°C for 1 min and 55°C for 1 min. Relative changes in mRNA or miRNA abundances were quantified by using the *C*_*t*_ method; *β*-actin mRNA and U6 RNA were used as reference amplicons for data normalization [25, 26].

## 3. Results and Discussions

### 3.1. mRNA Profile in Premetamorphosing and Prometamorphosing *P. olivaceus*

**Figure 1:**
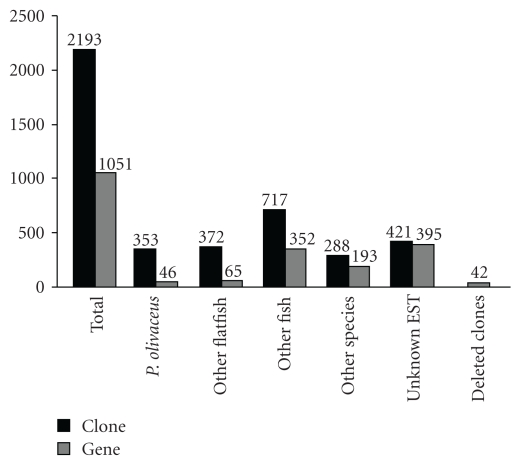
Summary of the EST distribution in various groups and the number of genes they represent. Solid bars are number of ESTs, and sketched bars are number of clones.

2235 clones were picked randomly from premetamorphosis and prometamorphosis cDNA library and sequenced. With exception of 42 empty clones, 2,193 cDNA clones were used to produce expressed sequence tags (ESTs) and represented 1,051 unique genes. Of 1,051 unique genes (GenBank Accession nos. GW882510-GW883514, GT229367-GT229408, and EU090804.1), only 46 unique genes (4.4%) were identified as homologous to the previously reported *P. olivaceus* genes, whereas 1005 (95.6%) unique genes were found to be novel *P. olivaceus* ESTs. Therefore, this EST collection represented a significant addition to the existing *P. olivaceus* EST resources. Of the new 1005 unique genes, 395 (39.3%) remained unknown in terms of their gene identity and others had the high number of BLASTX hits to fishes, including flatfish other than* P. olivaceus* (6.5%) and fishes other than flatfish (35.0%) (Figure 1).

**Figure 2:**
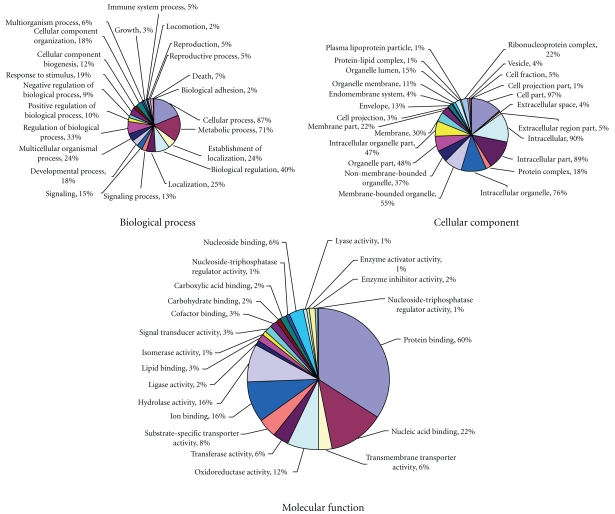
Gene Ontology (GO) assignment (2nd-level GO terms) of 1,825 annotated ESTs. The total numbers of ESTs annotated for each main category are 455 for Biological Process, 467 for Molecular Function, and 474 for Cellular Component. Since a gene product could be assigned to more than one GO term, the percentages in each main category do not add up to 100%.

Gene ontology (GO) categories were assigned to 656 unique ESTs using AmiGO database. The percentage distributions of gene ontology terms (2nd-level GO terms) according to the GO consortium are shown in Figure 2. Cellular Process (87%) was the most dominant 2nd-level term out of the 455 unique sequences which were annotated to the Biological Process GO category. This was followed by Metabolic Process Metabolism at 71%. It is noted that 9% were assigned to the Negative Regulation of Biological Process. Protein Binding (60%) was the most dominant out of 467 ESTs with significant protein hits which were assigned to Molecular Function category at 2nd level. This was followed by Nucleic Acid Binding at 20%. Cell Part (97%) was the most dominant out of 474 ESTs which were annotated to the Cellular Component GO category. Intracellular and intracellular parts occupied 90% and 89%, respectively. ESTs that fell in each of the three main GO categories are given in Figure 2.

**Figure 3:**
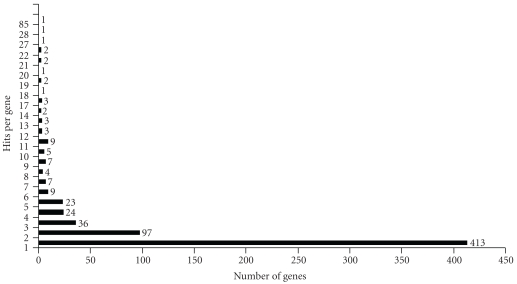
Expression profiles and sequencing redundancy of the known genes from the premetamorphosing and prometamorphosing *P. olivaceus*.

Compared with normalized cDNA library, the nonnormalized cDNA library is much more redundant. 2,193 cDNA clones from the nonnormalized cDNA library in this study only generated 1,051 unique genes. However, the nonnormalized cDNA library can provide raw information on the structure of gene expression level [27]. Among 656 identified distinct known genes in metamorphic *P. olivaceus* in this study, 413 known genes (63.0%) were sequenced only once, 180 genes (27.4%) were sequenced 2–5 times, and 63 genes (9.6%) were sequenced over 5 times. The vast majority of known genes were sequenced only once; however, a small number of genes accounted for a large proportion of transcripts in premetamorphosing and prometamorphosing *P. olivaceus* (Figure 3). The most abundantly expressed gene was parvalbumin accounting for 3.88% of the 2,193 clones sequenced (Table 3). The expressed gene beta-actin accounted for only 0.05%. The other most abundant expressed genes included cytochrome c oxidase subunit II (1.28%), ribosomal protein S2 (1.23%), cytochrome c oxidase subunit III (1.00%), creatine kinase 1 (1.00%), myosin light chain 3 (1.00%), 40S ribosomal protein S8 (1.00%), nuclease diphosphate kinase B (0.87%), ribosomal protein L18a (0.87%), and antifreeze protein type IV (0.87%). Altogether, the ten most abundantly expressed genes occupied 19.39% of all clones.

### 3.2. miRNA Profile in Premetamorphosing and Prometamorphosing *P. olivaceus*

**Figure 4:**
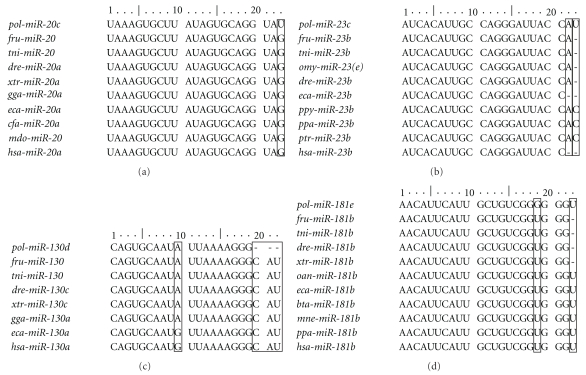
Alignment of the novel *P. olivaceus* miRNAs with highly preserved homologous miRNAs from other species.

MicroRNAs are small 19–23-nucleotide noncoding RNAs that bind to recognition sequences on 3′-untranslated regions (3′-UTRs) of mRNAs and target them for degradation or translational repression. MiRNAs have been found to play important roles in zebrafish development [28, 29]. miRNAs resources were developed only in few teleosts such as zebrafish, puffer fish, and *Oncorhynchus mykiss* [30–32]. No miRNAs have been identified in flatfish. In this study, total 143 clones picked randomly were sequenced (Table 4). Sequence analysis identified 29 microRNAs that showed the same as at least one published miRNAs in the database (http://www.mirbase.org/search.shtml). Representing 19 unique miRNAs are shown in Table 5. Four sequences had not been found to have the same sequences, but they showed significant similarities with published miRNAs in miRBase. In addition, there are four sequences identified as snoRNA by searching NCBI database. Overall, 23.08% of small RNAs in the library might be microRNAs and 2.80% were snoRNAs (Table 4). Names of the *P. olivaceus* miRNA were assigned based on the homologies between the cloned sequence and published miRNA sequences (Table 5). 19 unique miRNAs are conserved across several species and affiliated to 15 subfamilies, of which 9 unique miRNA (*pol-let-7a*, *pol-miR-7f*, *pol-miR-26a*, *pol-miR-125b*, *pol-miR-128*, *pol-miR-181a*, *pol-miR-200a*, *pol-miR-221*, and *pol-miR-429*) are conserved higher across ten or more species. The *pol-miR-125b* is conserved across 43 species. While mirRNAs, *pol-let-7j*, *pol-miR-21a*, *pol-miR-181f*, and *pol-miR-724*, are conserved across only one species (Table 5). Four miRNAs (*pol-miR-20c*, *pol-miR-23c*, *pol-miR-130d*, and *pol-miR-181e*) are novel to *P. olivaceus* characterized as having high homologies with published miRNAs but differed by at least one nucleotide. These 4 miRNAs only observed in *P. olivaceus* are of special interest because of their unique sequences and possibly unique targeting mechanisms (Figure 4). *Pol-miR-20c* has a U to G mismatch with miR-20 of *Fugu rubripes*, *Tetraodon nigroviridis,* and *Monodelphis domestica* or *miR-20a* of *Danio rerio*, *Xenopus tropicalis, Gallus gallus*, *Equus caballus*, *Canis familiaris,* and *Homo sapiens* (Figure 4(a)). *Pol-miR-23c* has a U to C mismatch at positions 23 with *miR-23b *of *Bos taurus*, *Pongo pygmaeus*, *Pan paniscus*, and *Pan troglodytes*. However, the position 23 is absent between *P. olivaceus* and other fishes. In the miR-23b of *E. caballus* and *H. sapiens*, the positions 23 and 22 are both absent (Figure 4(b)). *Pol-miR-130d* has an A to G mismatch at position 10 as compared with *E. caballus* and *H. sapiens*, whereas there is no mismatch at the position as compared with nonmammals. Compared with *miR-130* of other species, the positions 20, 21, and 22 of *P. olivaceus* are absent (Figure 4(c)). Compared with other species, *pol-miR-181e* has a G to U mismatch at the position 19. It is interesting that there is a one base absent at position 23 in other fishes and *Xenopus laevis*, whereas the position of U is conserved in *P. olivaceus* and higher vertebrates (Figure 4(d)).

### 3.3. The Expression Pattern of Representative Genes in Metamorphosing *P. Olivaceus*

**Figure 5:**
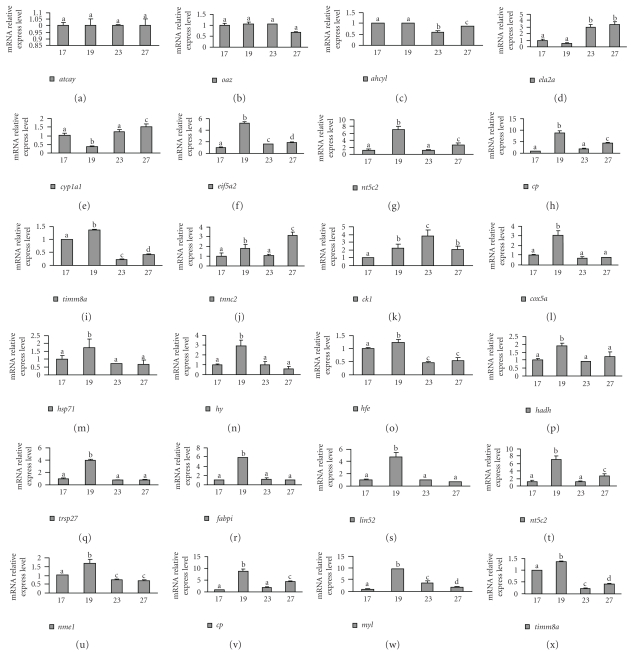
The abundance variety of mRNAs during metamorphic stage of *P. olivaceus*. The abundance of mRNAs was quantified by qRT-PCR. *β*-actin mRNA served as control for data normalization. Values are means ± SD, *n* = 3. Means without a common letter differ, *P* < .05.

Among miRNAs-targeted sequences *in silico* predicted by RNA22 miRNA target detection software [33], expression 24 genes of was confirmed in premetamorphosing or metamorphosing flounders by qRT-PCR. Only gene *atcay* was expressed stably at different metamorphic stages, indicating that it should not be associated with metamorphic events (Figure 5(a)). With exception for four genes *atcay*, *oaz*, *ahcyl*, and *ela2a* expression stably from premetamorphosis (17 DAH) to Stage E (19 DAH) (Figures 5(a)–5(d)), one gene *cyp1a1* expression decreased (Figure 5(e)) and other genes tested in this study had significantly increased expression level (Figures 5(f)– 5(x)), indicating that these genes might participate in early metamorphic events. After metamorphosis initiated (since 19 DAH), *oaz* expression level decreased (Figure 5(b)), and the expression level of genes *ahcyl*, *ela2a*, *cyp1a1*, *elf5a2*, *nt5c2*,* cp*, *timm8a*, and *tnnc2* or *ck1* fluctuated during metamorphosing stage (Figures 5(c)–5(k)), suggesting that these genes might be associated with later metamorphic events, whereas the expression level of gene *cox5a*,* hy*, *me*, *dck*, *mt-nd4l*, *hfe*, *hsp71*, *hadh*, *rps27*, *fabpi*, *lin52*, *nme1*, or *myl* during metamorphosing stage dropped down to the level at premetamorphosis stage (Figures 5(l)–5(x)), indicating that these genes might be unimportant for later metamorphic events.

**Figure 6:**
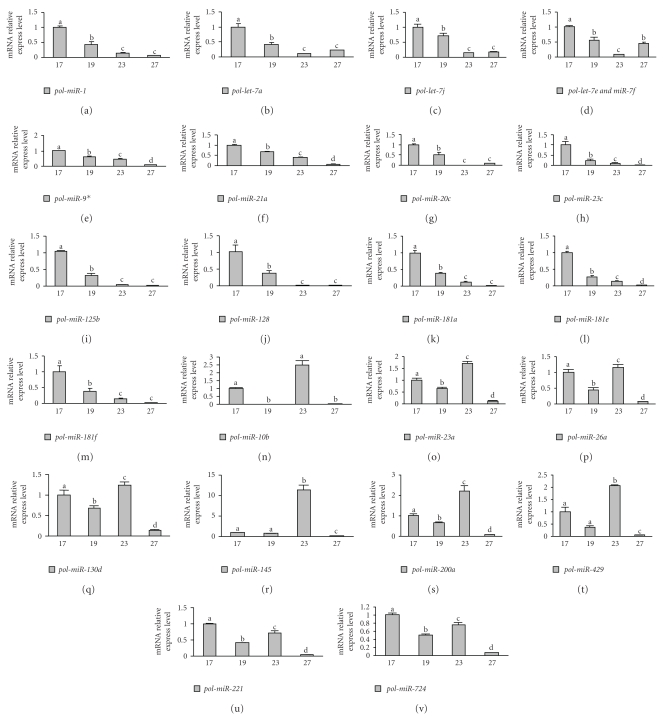
The abundance variety of miRNAs during metamorphic stage of *P. olivaceus*. The abundance of miRNAs was quantified by stem-loop qRT-PCR. U6 snRNA served as control for data normalization. Values are means ± SD, *n* = 3. Means without a common letter differ, *P* < .05.

To understand the role of miRNAs in the metamorphosing *P. olivaceus*, we quantified 23 miRNAs using stem-loop qRT-PCR. Since the sequence of *pol-miR-7e* is very similar to *pol-miR-7f*, the same pair of primers was used to amplify (Table 2). All 23 miRNAs were expressed in premetamorphosing or metamorphosing flounders. MicroRNAs *pol-miR-1*, *pol-miR-7a*, *pol-miR-7j*, *pol-miR-7e/7f*, pol-miR-9*, *pol-miR-21a*, *pol-miR-20c*, *pol-miR-23c*, *pol-miR-125b*, *pol-miR-128*, *pol-miR-181a*, *pol-miR-181e*, or *pol-miR-181f *were* e*xpressed highly just before metamorphosis starting (17 DAH), while their expression decreased after metamorphosis (from 19 DAH to 27 DAH), indicating that these microRNAs might not be associated with early metamorphic events (Figures 6(a)–6(m)). The expression level of miRNAs *pol-miR-10b*, *pol-miR-23a*,* pol-miR-26a*, *pol-miR-130d*, *pol-miR-145*, *pol-miR-200a*, *pol-miR-429*, *pol-miR-221*, or *pol-miR-724* fluctuated during metamorphosing stage, suggesting that they might be associated with metamorphosis (Figures 6(n)–6(v)). MicroRNAs *pol-miR-10b*, *pol-miR-23a*,* pol-miR-26a*, *pol-miR-130d*, *pol-miR-145*, *pol-miR-200a*, and *pol-miR-429 *were expressed at the highest level at 23 DAH and then decreased quickly (Figures 6(n)–6(t)), indicating that they might play roles in regulating metamorphosis at this stage. These results are consistent with the findings of previous studies demonstrating the importance of miRNAs in differentiation and development [28, 30].

## 4. Conclusion

In summary, we generated a collection of 1,051 unique ESTs, 23 unique miRNAs, and 4 unique snoRNAs in premetamorphosing and prometamorphosing* P. olivaceus*. Even though so far there were 3143 nucleotides and 13869 ESTs available in NCBI database, 1005 novel ESTs were identified successfully in this study, suggesting that special gene expression profile existed in metamorphic stage. Representative 24 mRNAs of 1051 unique ESTs were quantified during the metamorphosis of *P. olivaceus* using quantitative RT PCR, and the results showed that 20 genes might be associated with early metamorphic events and 10 genes might be related with later metamorphic events. In addition, the abundances of 23 miRNAs were quantified using stem-loop qRT PCR. 9 miRNAs might be associated with metamorphosis, and 7 miRNAs might play roles at metamorphic climax. The data provided in this research would be helpful for further identifying of metamorphosis-related genes in *P. olivaceus*.

## Figures and Tables

**Table 1:** Primers of mRNAs for qRT-PCR.

Clone name	Gene name	Primer name	Primer (5′-3′)
SFU-PO-DEV 0004	5-cytosolic ii (*nt5c2*)	CY2F	CGACCTACCTGCCAACATG
		CY2R	GTGCCAGACAACTGGTCC
SFU-PO-DEV 0125	cog1782: metal-dependent consists of a metallo-beta-lactamase domain and an rna-binding kh domain (*me*)	MEF	CTGACCAAACTGATCCGGC
		MER	CTGATCCAACATCGAGGTCG
SFU-PO-DEV 0133	Creatine kinase 1 mRNA (*ck1*)	CKF	GAGACTCGTGACTCTGCTCAC
		CKR	CAGCCTAGTGGAGGCTGATC
SFU-PO-DEV 0137	Cuticle protein (*cp*)	CP2F	GTGGCTACAACGCCGATG
		CPR	CAGGTGCTTTGTATGCAGGAG
SFU-PO-DEV 0159	Cytochrome c subunit va (*cox5a*)	CSF	GCAGCACTGAGAGCTTGTC
		CSR	GCTGCAGCTCTTGGATCAG
SFU-PO-DEV 0170	Deoxycytidine kinase (*dck*)	DK2F	GACTCCAAGCCCGGAACC
		DK2R	AGCTGAAGGCACAGCTAGTG
SFU-PO-DEV 0182	Elastase 2a (*ela2a*)	EL2F	GACCTGGCCAGTTGCAATG
		EL2R	GTAGTTGCAGCCCATGCTTG
SFU-PO-DEV 0200	Eukaryotic translation initiation factor 5A-2 (*eif5a2*)	ETF	CCATCGGCATCAAAGCCTTG
		ETR	GAGTAGCATTGACGAGGCAATG
SFU-PO-DEV 0213	Fatty acid binding protein intestinal (*fabpi*)	FAPF	CTATCAGGCTCGTGGACCATG
		FAPR	CTTTGCGTCCACACCTTCG
SFU-PO-DEV 0260	Heat shock protein 70 (*hsp71*)	HSPF	GCTACCTCCTGAGAAAGTGCTC
		HSPR	GTGACTCACTGCTCACTGAGTC
SFU-PO-DEV 0283	*Danio rerio* hypothetical LOC562892 (*hy*)	HYF	GCTGAGCTGCACTGATCAAG
		HYR	TGCTGCATGTGCACACTTG
SFU-PO-DEV 0295	Kiaa1872 protein (*atcay*)	KP2F	CGTCTTCCAGGCGACAG
		KP2R	GTACGGCCTGATGACCTG
SFU-PO-DEV 0297	l-3-hydroxyacyl-coenzyme ashort chain (*hadh*)	HCF	CAGAGTGAGCTGCTGAACAAG
		HCR	TGAAGTTCGGACTTGTCCCTC
SFU-PO-DEV 0298	Larval and non-ifm isoform (*myl*)	LIF	GCATGGATCCTGAAGATGACG
		LI2R	CAGGGTAAGGTCCAGCAATG
SFU-PO-DEV 0305	Lin-52 homolog (*lin52*)	LHF	GTTCTTCAAGTGGCGGGAC
		LHR	GCTGCAGTTCACTGTCTGAG
SFU-PO-DEV 0341	Middle subunit (ferritin m) (*hfe*)	FMF	GATTGAGCCTGAAGGGACGAG
		FMR	CGACACCAACACAACAGCTC
SFU-PO-DEV 0766	Nadh dehydrogenase subunit 4l (*mt-nd4l*)	NDF	CTCAGCCAACTTCTCAGCTTC
		NDR	GCTTTGAAGTCGGTCGGTAC
SFU-PO-DEV 0788	Nonmetastatic cells protein expressed in isoform a (*nme1*)	NMC-2F	GACTCCAAGCCCGGAACA
		NMC-2R	CAGTGGTCTCCGACCAGG
SFU-PO-DEV 0803	Ornithine decarboxylase antizyme small isoform (*oaz*)	ODAF	CGGGATCGCAATCTTTCAGC
		ODAR	GAGCAAGAAGCGCACTCTG
SFU-PO-DEV 0834	Putative cytochrome P450 like protein precursor (*cyp1a1*)	PCPF	GTCAAGCTCAACAGGCTCTTC
		PCPR	TGACGCGCATGAATGGATG
SFU-PO-DEV 0914	Ribosomal protein s27 (rps27)	RPF	CGGTTGATATCAGCGCCTTG
		RPR	CGATCTGTCAACGCGAACAG
SFU-PO-DEV 0945	s-adenosylhomocysteine hydrolase (*ahcyl*)	SAHF	GTACCTGGGTCTGCCCAG
		SAHR	CCAAACGCCAAACCCTTCTAG
SFU-PO-DEV 1002	Translocase of inner mitochondrial membrane 8 homolog a (*timm8a*)	TIMMF	TCCGAGGGCATGATGGAC
		TIMMR	GGACAGTTCTGCAACACTCAG
SFU-PO-DEV 1017	Troponin c type 2 (*tnnc2*)	TNNCF	CTACTGACCCCACTGTACCAC
		TNNCR	CCGTCTGTTGAGGATGTCAATG

**Table 2:** Primers of miRNAs for stem-loop qRT-PCR.

microRNA	Primer name	Primer (5′-3′)
	Universal revise primer	5*′*-GTGCAGGGTCCGAGGT
U6 snRNA	U6RT	5*′*-GTCAGGCAGCGTGCAGGGTCCGAGGTATTCGCACGCTGCCTGACAAAAAT
	U6F	5*′*-CGCAAGGATGACACGCAAATT
miR-1	1RT	5*′*-GTCGTATCCAGTGCAGGGTCCGAGGTATTCGCACTGGATACGACACATAC
	1F	5*′*-CGGCGGTGGAATGTAAAG
let-7a	7aRT	5*′*-GTCGTATCCAGTGCAGGGTCCGAGGTATTCGCACTGGATACGACAACTAT
	7aF	5*′*-CGGCGGTGAGGTAGTAGGTT
let-7e & miR-7f	7efRT	5*′*-GTCGTATCCAGTGCAGGGTCCGAGGTATTCGCACTGGATACGACAACTAT
	7efF	5*′*-CGGCGGTGAGGTAGTAGATT
let-7j	7jRT	5*′*-GTCGTATCCAGTGCAGGGTCCGAGGTATTCGCACTGGATACGACAACTGT
	7jF	5*′*-CGGCGGTGAGGTAGTTGTTT
miR-9*	9aRT	5*′*-GTCGTATCCAGTGCAGGGTCCGAGGTATTCGCACTGGATACGACACTTTC
	9aF	5*′*-CGGCGGTAAAGCTAGATAA
miR-10b	10bRT	5*′*-GTCGTATCCAGTGCAGGGTCCGAGGTATTCGCACTGGATACGACACAAAT
	10bF	5*′*-CGGCGGTACCCTGTAGAACC
miR-20c	20aRT	5*′*-GTCGTATCCAGTGCAGGGTCCGAGGTATTCGCACTGGATACGACATACCT
	20aF	5*′*-CGGCGGTAAAGTGCTTATAGT
miR-21	21RT	5*′*-GTCGTATCCAGTGCAGGGTCCGAGGTATTCGCACTGGATACGACCCAACA
	21F	5*′*-CGGCGGTAGCTTATCAGACT
miR-23a	23aRT	5*′*-GTCGTATCCAGTGCAGGGTCCGAGGTATTCGCACTGGATACGACTGGAAA
	23aF	5*′*-CGGCGGATCACATTGCCAGG
miR-23c	23bRT	5*′*-GTCGTATCCAGTGCAGGGTCCGAGGTATTCGCACTGGATACGACATGGTA
	23bF	5*′*-CGGCGGATCACATTGCCAGGG
miR-26a	26aRT	5*′*-GTCGTATCCAGTGCAGGGTCCGAGGTATTCGCACTGGATACGACAGCCTA
	26aF	5*′*-CGGCGGTTCAAGTAATCCAG
miR-125b	125bRT	5*′*-GTCGTATCCAGTGCAGGGTCCGAGGTATTCGCACTGGATACGACCACAAG
	125bF	5*′*-CGGCGGTCCCTGAGACCCT
miR-128	128RT	5*′*-GTCGTATCCAGTGCAGGGTCCGAGGTATTCGCACTGGATACGACAAAGAG
	128F	5*′*-CGGCGGTCACAGTGAACCG
miR-130d	130cRT	5*′*-GTCGTATCCAGTGCAGGGTCCGAGGTATTCGCACTGGATACGACCCCTTT
	130cF	5*′*-CGGCGGCAGTGCAATAT
miR-145	145RT	5*′*-GTCGTATCCAGTGCAGGGTCCGAGGTATTCGCACTGGATACGACGGGATT
	145F	5*′*-CGGCGGGTCCAGTTTTCCCA
miR-181a	181aRT	5*′*-GTCGTATCCAGTGCAGGGTCCGAGGTATTCGCACTGGATACGACACTCAC
	181aF	5*′*-CGGCGGAACATTCAACGCTGT
miR-181e	181a-1RT	5*′*-GTCGTATCCAGTGCAGGGTCCGAGGTATTCGCACTGGATACGACAACTCA
	181a-1F	5*′*-CGGCGGAACATTCAACGCTGTC
miR-181f	181fRT	5*′*-GTCGTATCCAGTGCAGGGTCCGAGGTATTCGCACTGGATACGACACCCCC
	181fF	5*′*-CGGCGGAACATTCATTGCTGT
miR-200a	200aRT	5*′*-GTCGTATCCAGTGCAGGGTCCGAGGTATTCGCACTGGATACGACACATCG
	200aF	5*′*-CGGCGGTAACACTGTCTGGT
miR-221	221RT	5*′*-GTCGTATCCAGTGCAGGGTCCGAGGTATTCGCACTGGATACGACGAAACC
	221F	5*′*-CGGCGGAGCTACATTGTCTGC
miR-429	429RT	5*′*-GTCGTATCCAGTGCAGGGTCCGAGGTATTCGCACTGGATACGACACGGCA
	429F	5*′*-CGGCGGTAATACTGTCTGGT
miR-724	724RT	5*′*-GTCGTATCCAGTGCAGGGTCCGAGGTATTCGCACTGGATACGACAACAGT
	724F	5*′*-CGGCGGTTAAAGGGAATTTG

**Table 3:** The ten most highly expressed genes in premetamorphosing and prometamorphosing *P. olivaceus*.

Putative identification	Frequency (%)
Parvalbumin	3.88%
Cytochrome c oxidase subunit II	1.28%
Ribosomal protein S2	1.23%
Cytochrome c oxidase subunit III	1.00%
Creatine kinase 1	1.00%
Myosin light chain 3	1.00%
40S ribosomal protein S8	1.00%
Nuclease diphosphate kinase B	0.87%
Ribosomal protein L18a	0.87%
Antifreeze protein type IV	0.87%

**Table 4:** Classification of cloned small RNAs from *P. olivaceus*.

RNA species	Number present	% of Total clones
miRNAs shown in Table 5 ^a^	29	20.28%
Novel miRNAs shown in Figure 4 ^b^	4	2.80%
rRNA	21	14.68%
snoRNA	4	2.80%
Unidentified^c^	45	31.47%
Low quality or short sequences^d^	40	27.97%

Total	143	100%

^a^Only miRNA candidates that match 100% to one of miRNAs in the miRBase database.

^b^MiRNA candidates without 100% match to any miRNAs in the miRBase database, but with significant similarity

^c^Sequences do not match any known miRNAs or any other small RNAs or mRNAs.

^d^Low-quality sequences and sequences less than 17 nucleotides were not analyzed further.

**Table 5:** Sequence and characteristics of conserved miRNAs in *P. olivaceus*.

miRNA family	miRNA name	No. of clones	Sequence (5′-3′)	Length (bp)	Conserved in other animals
pol-miR-1	pol-miR-1a	1	UGGAAUGUAAAGAAGUAUGUA	21	ssc, cfa, mmu-miR-1-2-as, mdo, xtr, ppa, gga, cbr, cel
pol-let-7	pol-let-7a	3	UGAGGUAGUAGGUUGUAUAGUU	22	eca, bfl-let-7-1-as, sko, lgi, bfl, ptr, cfa, mml, mdo, bta, xtr, tni, fru, dre, gga, rno, mmu, cbr, hsa, cel
	pol-let-7e	2	UGAGGUAGUAGAUUGAAUAGUU	22	oan, tni, fru, dre, gga
	pol-miR-7f	1	UGAGGUAGUAGAUUGUAUAGUU	22	eca, ptr, cfa, mml, oan, mdo, xtr, bta, ssc, dre, gga, rno, mmu,
	pol-let-7j	1	UGAGGUAGUUGUUUGUACAGUU	22	dre
pol-miR-9*	pol-miR-9*	3	UAAAGCUAGAUAACCGAAAGU	21	mdo, xtr, dre
pol-miR-10	pol-miR-10b	1	UACCCUGUAGAACCGAAUUUGU	22	oan, xtr, ppa, mne, ggo, gga, omy
pol-miR-21	pol-miR-21a	1	UAGCUUAUCAGACUGGUGUUGG	22	omy
pol-miR-23	pol-miR-23a	3	AUCACAUUGCCAGGGAUUUCCA	22	oan, bta, tni, fru, dre
pol-miR-26	pol-miR-26a	1	UUCAAGUAAUCCAGGAUAGGCU	22	eca, cfa, bta, oan, tni, fru, ppa, mml, mne, lla, ppy, ggo, ptr, ssc, dre, rno, mmu, hsa, omy
pol-miR-125	pol-miR-125b	1	UCCCUGAGACCCUAACUUGUGA	22	eca, bfl, spu, sko, cap, bfl, dya, dwi, dvi, dsi, dse, dpe, dmo, dgr, der, dan, tca, cfa, oan, mdo, xtr, bta, tni, fru, lca, mne, lla, sla, mml, ptr, ppy, ppa, age, ggo, ssc, dre, aga, dps, gga, rno, hsa, dme, mmu
pol-miR-128	pol-miR-128	2	UCACAGUGAACCGGUCUCUUU	21	eca, cfa, mml, oan, mdo, bta, ptr, gga, rno, hsa, ppa, mmu, omy
pol-miR-145	pol-miR-145	1	GUCCAGUUUUCCCAGGAAUCCC	22	dre, omy
pol-miR-181	pol-miR-181a	1	AACAUUCAACGCUGUCGGUGAGU	23	eca, oan, mdo, xtr, tni, fru, lla, ppy, mne, sla, mml, ptr, ppa, ggo, dre, gga, rno, hsa, mmu, omy
	pol-miR-181f	1	AACAUUCAACGCUGUCGGUGAGUU	24	bta
pol-miR-200	pol-miR-200a	1	UAACACUGUCUGGUAACGAUGU	22	eca, ptr, mml, mdo, xtr, tni, fru, dre, gga, rno, hsa, mmu
pol-miR-221	pol-miR-221	1	AGCUACAUUGUCUGCUGGGUUUC	23	eca, ptr, mdo, xtr, tni, fru, ppa, ppy, ggo, mml, dre, gga, rno, mmu, hsa
pol-miR-429	pol-miR-429	3	UAAUACUGUCUGGUAAUGCCGU	22	bta, oan, xtr, gga, tni, fru, dre, cfa, rno, mmu
pol-miR-724	pol-miR-724	1	UUAAAGGGAAUUUGCGACUGUU	22	dre

## References

[1] Hashimoto H, Mizuta A, Okada N (2002). Isolation and characterization of a Japanese flounder clonal line, reversed, which exhibits reversal of metamorphic left-right asymmetry. *Mechanisms of Development*.

[2] Inui Y, Miwa S (1985). Thyroid hormone induces metamorphosis of flounder larvae. *General and Comparative Endocrinology*.

[3] Miwa S, Inui Y (1987). Effects of various doses of thyroxine and triiodothyronine on the metamorphosis of flounder (*Paralichthys olivaceus*). *General and Comparative Endocrinology*.

[4] Schreiber AM, Specker JL (1998). Metamorphosis in the summer flounder *Paralichthys dentatus*: stage- specific developmental response to altered thyroid status. *General and Comparative Endocrinology*.

[5] Soffientino B, Specker JL (2001). Metamorphosis of summer flounder, *Paralichthys dentatus*: cell proliferation and differentiation of the gastric mucosa and developmental effects of altered thyroidal status. *Journal of Experimental Zoology*.

[6] Yamano K, Miwa S (1998). Differential gene expression of thyroid hormone receptor *α* and *β* in fish development. *General and Comparative Endocrinology*.

[7] Kopchick JJ, Andry JM (2000). Growth hormone (GH), GH receptor, and signal transduction. *Molecular Genetics and Metabolism*.

[8] Bao B, Yang G, Liu Z, Li S, Wang Z, Ren D (2005). Isolation of SFRS3 gene and its differential expression during metamorphosis involving eye migration of Japanese flounder *Paralichthys olivaceus*. *Biochimica et Biophysica Acta*.

[9] Hildahl J, Sweeney G, Galay-Burgos M, Einarsdóttir IE, Björnsson BT (2007). Cloning of Atlantic halibut growth hormone receptor genes and quantitative gene expression during metamorphosis. *General and Comparative Endocrinology*.

[10] Kono T, Sakai M (2001). The analysis of expressed genes in the kidney of Japanese flounder, *Paralichthys olivaceus*, injected with the immunostimulant peptidoglycan. *Fish and Shellfish Immunology*.

[11] Clark MS, Edwards YJK, Peterson D (2003). Fugu ESTs: new resources for transcription analysis and genome annotation. *Genome Research*.

[12] Rise ML, von Schalburg KR, Brown GD (2004). Development and application of a salmonid EST database and cDNA microarray: data mining and interspecific hybridization characteristics. *Genome Research*.

[13] Wang S, Peatman E, Abernathy J (2010). Assembly of 500,000 inter-specific catfish expressed sequence tags and large scale gene-associated marker development for whole genome association studies. *Genome Biology*.

[14] Rexroad CE, Lee Y, Keele JW (2003). Sequence analysis of a rainbow trout cDNA library and creation of a gene index. *Cytogenetic and Genome Research*.

[15] Li P, Peatman E, Wang S (2007). Towards the ictalurid catfish transcriptome: generation and analysis of 31,215 catfish ESTs. *BMC Genomics*.

[16] Xu B, Wang S, Jiang Y (2010). Generation and analysis of ESTs from the Grass carp, *Ctenopharyngodon idellus*. *Animal Biotechnology*.

[17] Johnston RJ, Hobert O (2003). A microRNA controlling left/right neuronal asymmetry in *Caenorhabditis elegans*. *Nature*.

[18] Miwa S, Inui Y (1987). Histological changes in the pituitary-thyroid axis during spontaneous and artificially-induced metamorphosis of larvae of the flounder *Paralichthys olivaceus*. *Cell and Tissue Research*.

[19] Miwa S, Tagawa M, Inui Y, Hirano T (1988). Thyroxine surge in metamorphosing flounder larvae. *General and Comparative Endocrinology*.

[20] Conesa A, Götz S, García-Gómez JM, Terol J, Talón M, Robles M (2005). Blast2GO: a universal tool for annotation, visualization and analysis in functional genomics research. *Bioinformatics*.

[21] Ashburner M, Ball CA, Blake JA (2000). Gene ontology: tool for the unification of biology. *Nature Genetics*.

[22] Vizcaíno JA, González FJ, Suárez MB (2006). Generation, annotation and analysis of ESTs from Trichoderma harzianum CECT 2413. *BMC Genomics*.

[23] Ambros V, Bartel B, Bartel DP (2003). A uniform system for microRNA annotation. *RNA*.

[24] Chen C, Ridzon DA, Broomer AJ (2005). Real-time quantification of microRNAs by stem-loop RT-PCR. *Nucleic Acids Research*.

[25] Livak KJ, Schmittgen TD (2001). Analysis of relative gene expression data using real-time quantitative PCR and the 2T method. *Methods*.

[26] Schmittgen TD, Livak KJ (2008). Analyzing real-time PCR data by the comparative C(T) method. *Nature Protocols*.

[27] Hackett JD, Scheetz TE, Yoon HS (2005). Insights into a dinoflagellate genome through expressed sequence tag analysis. *BMC Genomics*.

[28] Giraldez AJ, Mishima Y, Rihel J (2006). Zebrafish MiR-430 promotes deadenylation and clearance of maternal mRNAs. *Science*.

[29] Morton SU, Scherz PJ, Cordes KR, Ivey KN, Stainier DYR, Srivastava D (2008). microRNA-138 modulates cardiac patterning during embryonic development. *Proceedings of the National Academy of Sciences of the United States of America*.

[30] Chen PY, Manninga H, Slanchev K (2005). The developmental miRNA profiles of zebrafish as determined by small RNA cloning. *Genes and Development*.

[31] Ramachandra RK, Salem M, Gahr S, Rexroad CE, Yao J (2008). Cloning and characterization of microRNAs from rainbow trout (*Oncorhynchus mykiss*): their expression during early embryonic development. *BMC Developmental Biology*.

[32] Salem M, Xiao C, Womack J, Rexroad CE, Yao J (2009). A microRNA repertoire for functional genome research in rainbow trout (*Oncorhynchus mykiss*). *Marine Biotechnology*.

[33] Miranda KC, Huynh T, Tay Y (2006). A pattern-based method for the identification of microRNA binding sites and their corresponding heteroduplexes. *Cell*.

